# Semiochemicals released from five bacteria identified from animal wounds infested by primary screwworms and their effects on fly behavioral activity

**DOI:** 10.1371/journal.pone.0179090

**Published:** 2017-06-08

**Authors:** Junwei J. Zhu, Muhammad F. Chaudhury, Lisa M. Durso, Agustin Sagel, Steven R. Skoda, Nadia S. Jelvez-Serra, Euzebio Goulart Santanab

**Affiliations:** 1 USDA-ARS, Agroecosystem Management Research Unit, East Campus, UNL, Lincoln, Nebraska, United States of America; 2 USDA-ARS, Screwworm Research Unit, UNL-EC, Lincoln, Nebraska, United States of America; 3 USDA-ARS, Screwworm Research Unit, Pacora, Republic of Panama; 4 USDA-ARS, Screwworm Research Unit, Knipling-Bushland U. S. Livestock Insects Research Laboratory, Kerrville, Texas, United States of America; 5 Instituto de Quimica e Biotecnologia, Universidade Federal de Alagoas, Maceió, Alagoas, Brazil; Gaziosmanpasa Universitesi, TURKEY

## Abstract

**Background:**

The Primary screwworm, *Cochliomyia hominivorax* (Coquerel), is a serious pest feeding on living flesh of any warm-blooded animal, including humans. It was eradicated from the United States in the early 1980s using the sterile male technique. However, it was recently detected in populations of wild deer and pets in the Florida Keys of the US. For monitoring purposes, screwworm flies are normally trapped using attractant bait with liver. However, there has been little effort to develop an efficient monitoring system for detection of screwworm flies using a specific synthetic attractant blend. Several studies have shown that odors from animal wound fluids attract screwworm adults, particularly gravid females. Bacteria associated with animal wounds have been identified that act as a major source for this attraction. To understand what volatiles attract screwworms we inoculated bovine blood with previously identified bacteria. We identified volatile chemicals released from the inoculated blood and other selected media over time and assessed the effect of those chemicals on behavioral activity of adult screwworm flies.

**Methodology/Principal findings:**

A total of 7 volatile compounds were collected from bacteria incubated in either broth or blood using solid-phase microextraction, and their chemical structures were identified by their characteristic mass spectrum fragments and confirmed by retention times in comparison to those of synthetic standards via gas chromatograph combined mass spectrometry analyses. Five major volatiles including dimethyl disulfide, dimethyl trisulfide, phenol, p-cresol and indole were detected from a mixture of 5 bacteria incubated in blood. The ratios of volatiles released differed among different incubation media, time and individual bacteria. A synthetic mixture containing the five compounds was demonstrated to be attractive to adult screwworm flies both in laboratory assays and field trapping trials.

**Conclusions/Significance:**

The results obtained from this study may assist in developing an efficient trapping system using the identified attractant blend to detect the infestation of primary screwworms. This is also the first study to explore the complex systems in volatile release profiles from 5 bacteria isolated from screwworm-infested animal wounds that are incubated with different media and incubation time, as well as individual and multi-species bacterial communities.

## Introduction

The Primary screwworm (also known as the New World screwworm), *Cochliomyia hominivorax* (Coquerel), is found in the Western Hemisphere, primarily in tropical areas of South America and some Caribbean Islands. The larvae of screwworms feed on the living flesh of warm-blooded animals including humans [1–3]. After their entry into wounds or body orifices, they start feeding on living tissue. If untreated, screwworm infestations can be fatal [4,5]. Screwworm was eradicated from the United States in the early 1980s, largely due to the success of the sterile male technique [6]. However, Primary screwworm infestations were recently detected in populations of wild deer and pets in the Florida Keys of the U.S. [7].

Guillot et al. [8,9] discovered that 96% of screwworm flies that visited wounds were inseminated, which suggested that wounds may be associated with feeding and oviposition. The vitellogenic stage of development was found to be highly variable indicating that some made visits for reasons other than oviposition [10]. Guillot et al. [9] suggested that wound fluids are an important source of nutrition for female *C*. *hominivorax*, and Thomas & Mangan [11] further demonstrated that average feeding visit time of individual females was 5.4 min, which was much longer than male flies. Screwworm flies have been reported to be attracted to odors from decomposing meat and liver [2,12], spent artificial diet from larval rearing [13,14], and cultures from a variety of bacterial species [15]. DeVaney et al. [16] and Eddy et al. [15] further showed that bacteria and/or compounds produced by them were the source of odors in incubated blood that were attractive to gravid screwworm flies. A number of bacterial species associated with screwworm fly attraction and oviposition have been isolated [15–19]. Chaudhury et al. [14, 20] suggested that volatile releases of at least 5 of 8 wound-isolated bacteria species that were incubated with bovine blood may play important roles in attracting gravid screwworms.

In the present study, we investigated 1). What volatiles were released from the five screwworm wound-associated bacterial species inoculated in different culturing media (broth and blood); 2). Different release profiles of volatiles from a mixture of five bacteria and individual bacteria species in blood media; 3). Differences in released volatiles through various incubation periods, and 4). The effect of the volatiles on behavioral activities in screwworm attraction and oviposition preferences.

## Materials and methods

### Ethics statement

The primary screwworm flies used for the present study are insect pests; they are not on the list of endangered and protected species. The collection of volatiles from bacteria incubated in bovine blood was conducted in a BSL-2 laboratory following Federal Safety Protocols.

### Insects

Primary screwworms were reared in the facility of the USDA-ARS BSL-2 laboratory in Pacora, Panama on a cellulose-based artificial diet as described in Chaudhury et al. (2007). The adults were maintained at 25 ± 1°C, 50 ± 5% RH, and a photoperiod of 12:12h (L:D) as described by Chaudhury et al. [19]. Flies were separated by sex on the day they emerged from puparia. Ten-day-old gravid females were used for the oviposition assay.

### Bacteria

Five bacteria spp. were isolated from screwworm-infested animal wounds as reported in Chaudhury et al. [19], and these wild types of bacteria species were identified using the API-20E enteric identification system (bio-Merieux, S.A., Marcy, LÕEtoile, France; bio-Merieux, Inc., Durham, NC). They were all gram-negative coliform rod-shaped bacteria. The five bacteria used for the current study were the same species as identified in Chaudhury et al. [19], but with the strains ordered from ATCC as follows: *Klebsiella oxytoca* (Flugge) ATCC^®^ 51817^™^, *Proteus mirabilis* Hauser ATCC^®^ 212721^™^, *Proteus vulgaris* Hauser ATCC^®^ 9920^™^, *Providencia rettgeri* Rettger ATCC^®^ 9918^™^ and *Providencia stuartii* Ewing ATCC^®^ 35031^™^.

### Blood

Fresh bovine blood was obtained aseptically from the Meat Animal Research Center (MARC) Animal Facility in Clay Center, Nebraska, and treated with EDTA as anticoagulant at the rate of 2 g/liter blood. Blood was refrigerated at 5°C until use.

### Preparation of Blood/Broth-Bacteria samples

The five bacterial isolates used for this study were obtained from the American Type Culture Collection, Manassas, VA. Bacteria were grown overnight at 37°C in Tryptic Soy Broth. Each bacteria culture was then adjusted to a common OD_600_ of 0.3 corresponding to approximately 10^8^ cfu/ml (means of 3 replicates) and stored on ice before being inoculated into sterile bovine blood. Plate counts were made to confirm OD_600_ readings. All five bacteria are members of the family *Enterobacteriaceae*, and are expected to have similar replication times. Species-level growth curves were not performed as part of these experiments, so the final concentration of each strain relative to the others at the final time point is unknown. For collection of volatiles, 1 ml of each individual or combined 5 bacteria culture was mixed with 9 ml of either blood or broth into a 20 ml vial. Vials were covered with caps and incubated at 37°C for 24, 48, 72, 96 and 120 hours in separate batches and used immediately for volatile collection. Bacteria are expected to be in late log/early stationary phase at the 24-hour time point, and at stationary phase for the remaining time points. Each replicate contained 6 vials, with 3 bacteria-inoculated and 3 controls (without bacteria added). A total of 3 replicates were conducted. Control samples were prepared, incubated and handled the same way as the bacteria ones. Volatile collection using solid phase microextraction method lasted up to 8 hours.

### Volatile collection and chemical compound identification

Volatiles from either bacteria-inoculated blood samples or controls were collected by solid-phase microextraction (SPME). We used a SPME portable field sampler with a Carboxen/PDMS fiber (75 mm carboxen / polydimethylsiloxane; Supelco, Bellefonte, PA, USA). The SPME sampler was preconditioned for 1 h at 300°C in the injection port of a gas chromatograph. For each sample, the SPME fiber was placed ≈2.0 cm above the top surface of the blood sample (stationary phase) for up to 8 hours, which the absorption of most of released odorant compounds to SPME fiber reached an equilibrium time for quantitative analysis [21, 22]. Each SPME sampler was designated for collecting odors from a single vial throughout the 4-day collection period.

Volatiles absorbed on SPME fibers were thermo-desorbed at 200°C and analyzed with a gas chromatography (GC)-mass spectrometry (MS) system equipped with DB-Wax or FFAP columns (30 m × 0.25 mm diameter, with 0.25 μm film thickness; Agilent Inc., Palo Alto, CA). Helium was used as the carrier gas at a flow rate of 1.5 ml/min. Samples were injected under the paused-splitless mode with a temperature program of 50°C for 3 min, rising by 10°C/min to 240°C. Compounds were identified by comparison of retention times and mass spectra with those of synthetic standards using Wiley MS Library Database. After each analysis, the SPME was cleaned by placing it in the heated GC injection port at 250°C for 5 min. The cleaned fiber was reanalyzed in the GC to ensure that no material was retained prior to reuse. Synthetic standards of the identified volatile compounds from all samples were purchased from Sigma-Aldrich (St. Louis, Missouri, USA), and the label purity of these chemicals was >95%.

Volatiles adsorbed to SPME fibers were quantified using linear calibration curves for each identified compound developed using five concentrations (5, 10, 50, 100, and 500 ng/μl) with three replications per concentration. Linearity was accepted when linear regression R^2^ > 0.96. The compounds were quantified by integrating peak areas with the calibrated curves.

### Preparations of synthetic attractant blends for behavioral tests

Synthetic blends containing the five detected compounds, dimethyl disulfide (DMDS), dimethyl trisulfide (DMTS), phenol, p-cresol, and indole (> 95% purity) dissolved in ethanol were used in laboratory and field behavioral experiments. Each blend contained approximately 600 mg of compounds (in 1 ml solution) at the ratio adjusted to match the SPME volatile collection.

### Behavioral responses and oviposition assays

Behavioral responses of screwworm flies to the synthetic blend of 5 compounds dissolved in 1 ml of ethanol were tested at three dosages of 600 mg, 60 mg and 6 mg in locally-fabricated metal wire mesh cages (0.5x0.5x0.5m, with solid metal base and the front opening secured by a stretched cotton polyester sleeve) under laboratory conditions (23 ± 2°C and 50% R.H.) as described by Chaudhury et al. [14]. A total of one hundred 7-day old male and female screwworm flies (50 each sex) were released into the metal wire cage. Two 9-cm trapping disks cut out from yellow sticky trap material (Great Lakes IPM Inc., Vestaburg, Michigan, USA) with a cotton wick (Crosstex International Inc., New York, USA) in the center of the disk were placed in the cage. On one disk the 1cm long cotton wick was baited with 1ml of synthetic blends and the other with 1 ml ethanol (control). The treatment disk and the control disk were placed about 8 cm apart. After ½ hour, the flies caught on the surface of the disks were counted and separated by sex. A total of three replicates were conducted for each dosages tested. Treatments were randomly assigned to the trapping disks.

Oviposition tests were conducted in similar cages described above but smaller in size (0.3x0.3x0.3m) using fifty 10 day old gravid females per cage. Females were transferred from the colony cage using a 20 cm x 1 cm diameter glass tube aspirator (without CO_2_ or cold anesthesia) to each test cage through the front opening that was secured with a stretched cotton/polyester sleeve. A pair of disposable Petri dishes (10 cm in diameter) was used to prepare the oviposition substrate test samples, each with 5 g of fresh ground beef on the center of the dish. A 1 cm long dental wick treated with 0.5 ml of the synthetic blend was placed on top of the ground beef in one of the dishes. The second dish received a similar dental wick with equal volume of ethanol as control. Each pair of Petri dishes was then introduced in each test cage and placed on the base of the cage, one at each diagonal corner. Flies were given the opportunity to oviposit for 1 h when the cage was kept at 35°C and 60% RH in complete darkness to prevent visual orientation to the substrates. After 1 h, the Petri dishes were removed from the cage, the egg masses in each dish were collected, and the weight of eggs per dish was recorded. A total of at least 6 replicates were conducted for each oviposition assay.

### Field trapping trials

Field trials to test whether the identified blend was attractive to primary screwworm flies were conducted in a hog farm (Povoado Santa Candida) in Penedo, Brazil, in which no insecticides were used on the animals or the surroundings. The trap was made from a 5-gallon black plastic bucket bottom, with a diameter of 40 cm, purchased from a Menard store in Lincoln, Nebraska, USA. A 6-cm hole, cut at each traps’ center, was used to hold a thin metal wire to which was attached a dental wick impregnated with the identified attractant blend (1ml). Three dosages of the blend at 3 g (5x), 600 mg (1x) and 60 mg (1/10x) were tested. Control baits contained ethanol only (1 ml). The surface of the trap was evenly applied with insect sticky glue (Tanglefoot Inc. USA). The trap was hung from the roof above (approximately 1 m) the animals. The trap positions were randomized, and the minimum distance between the two traps was at least 7–10 m. The trapping experiments started at 08:30 and were left for 48 hours before lures were replaced. After counting captured flies on each trap the flies were carefully removed, using forceps. Flies were brought back to the laboratory and identified as screwworms and were separated as males and females according to their morphological characteristics [23]. A total of 6 replicates were conducted.

#### Statistical analysis

The significance of differences from field and indoor behavioral responses to the identified blend at different dosages as well as amount of eggs laid among synthetic attractant blends of different dosages were determined by either Student T-test or One-way ANOVA followed by LSD test (PASW Statistics 18, SPSS Inc. Released 2009. SPSS Statistics for Window, Version 18.0 Chicago: SPSS Inc.) Different letters above bars indicate statistical significance at *p* < 0.05.

## Results and discussion

### Differences in identified volatiles released from media

**Fig 1:**
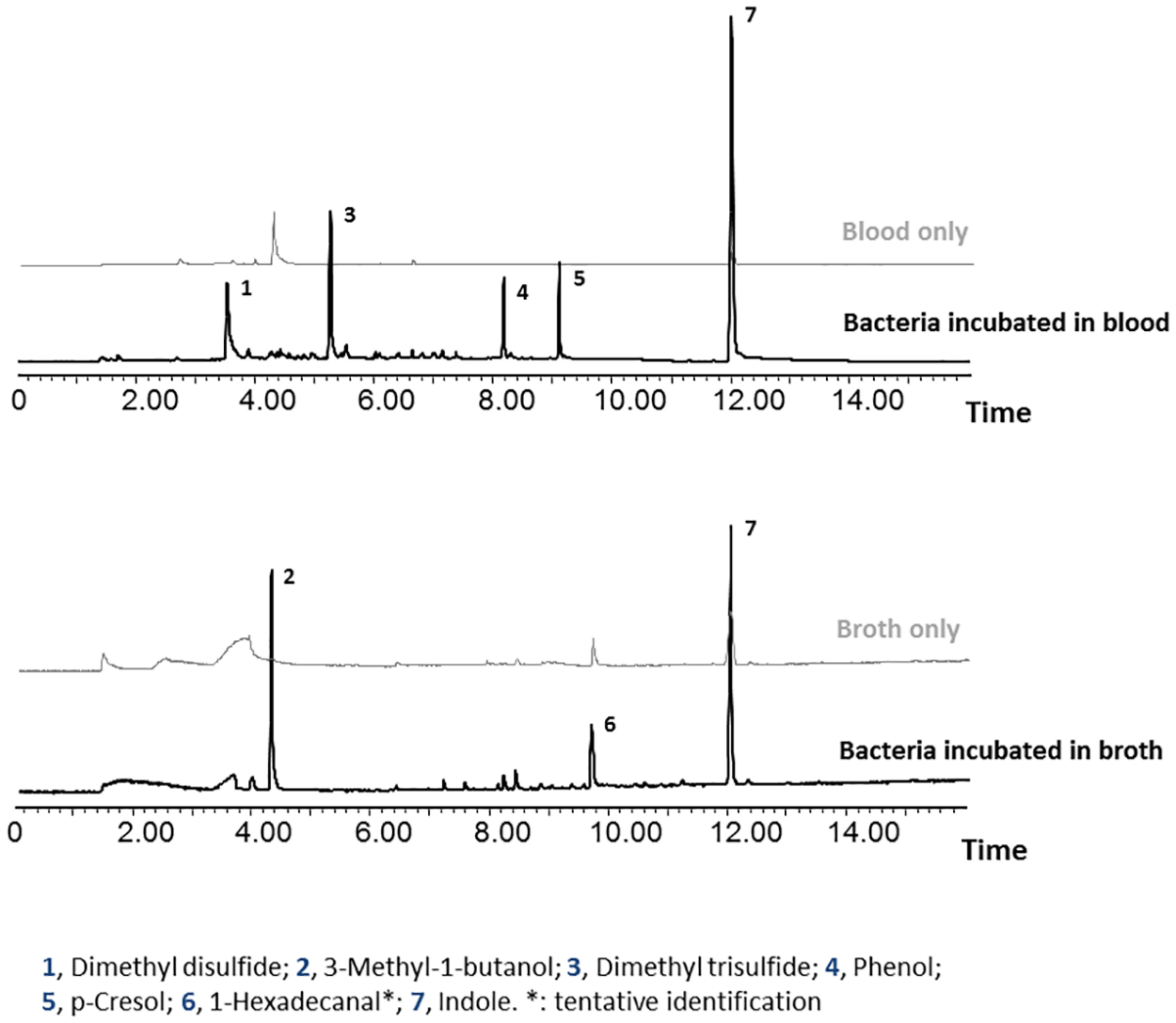
Solid phase micro-extraction absorptions of volatiles released from bacteria incubated with blood and broth for 72 hours, as well as their controls with medium only (8 hours collection).

A total of 7 volatile compounds were identified from bacteria incubated with either broth or blood (Table 1). All of these volatile compounds have been reported as the most widespread volatile aromatic compounds produced by phylogenetically diverse bacteria [24]. Table 1 represents the amounts of those absorbed on the SPME fibers and consistently detected. No odorant compounds were detected from either blood or broth alone, with exception of trace amount of indole found from both media after 8 hours of SPME collection (Fig 1). From collections of a mixture of 5 bacteria incubated in blood, five major volatiles including dimethyl disulfide, dimethyl trisulfide, phenol, p-cresol and indole were detected. Some of these compounds were previously identified from primary screwworm larvae infested animal wound fluid [25, 26]. However, only three volatiles, 3-methyl-1-butanol, 1-hexadecanal and indole, were identified from inoculated broth. Similar results have been reported that different volatile compounds were released from the apple maggot uricase (+) strain of *Pantoea* (*Enterobacter) agglomerans* (Ewing and Fife) cultured on media that differed in their primary source of nutrients [27].

**Table 1:** Hourly absorption rates (ng) of solid phase microextraction of volatiles released from five bacteria incubated with blood or broth for 72 hours).

	Incubation media used* (Means ± Standard Errors)	
Compounds	Blood	Broth
Dimethyl disulfide	1.73 ± 0.63	---
3-Methyl butanol	---	0.24 ± 0.05
Dimethyl trisulfide	2.17 ± 0.81	---
Phenol	1.55 ± 0.33	---
*p*-Cresol	0.98 ± 0.40	---
1-Hexadecanal	---	0.15 ± 0.02
Indole	3.15 ± 1.10	1.24 ± 0.59

* Presented here were SPME fiber-absorption rates of collected volatiles released from bacteria inoculated and incubated media for 72 hours.

Quite a variety of volatile compounds have been reported as bacterial metabolites including carboxylic acids, alcohols, aldehydes, esters, hydrocarbons and organic sulfur derivatives [24]. Among five volatiles identified from the present study, volatile sulfur compounds such as dimethyl disulfide and dimethyl trisulfide are degradation products of sulfur-containing materials that contribute to the sulfur cycle [28]. These two compounds have also been reported to have been released from headspace collection of *Pseudomonas* and *Enterobacter* bacteria species [29]. Phenol has been identified from volatiles released from *Klebsiella pneumoniae* (Schroeter) and *Citrobacter freundii* (Braak) [27, 30, 31]. Cresol is also a metabolite of a marine Arctic bacterium from the Cytophaga-Flavobacterium-Bacteroides group [32], as well as from the cyanobacterium *Calothrix*.

**Fig 2:**
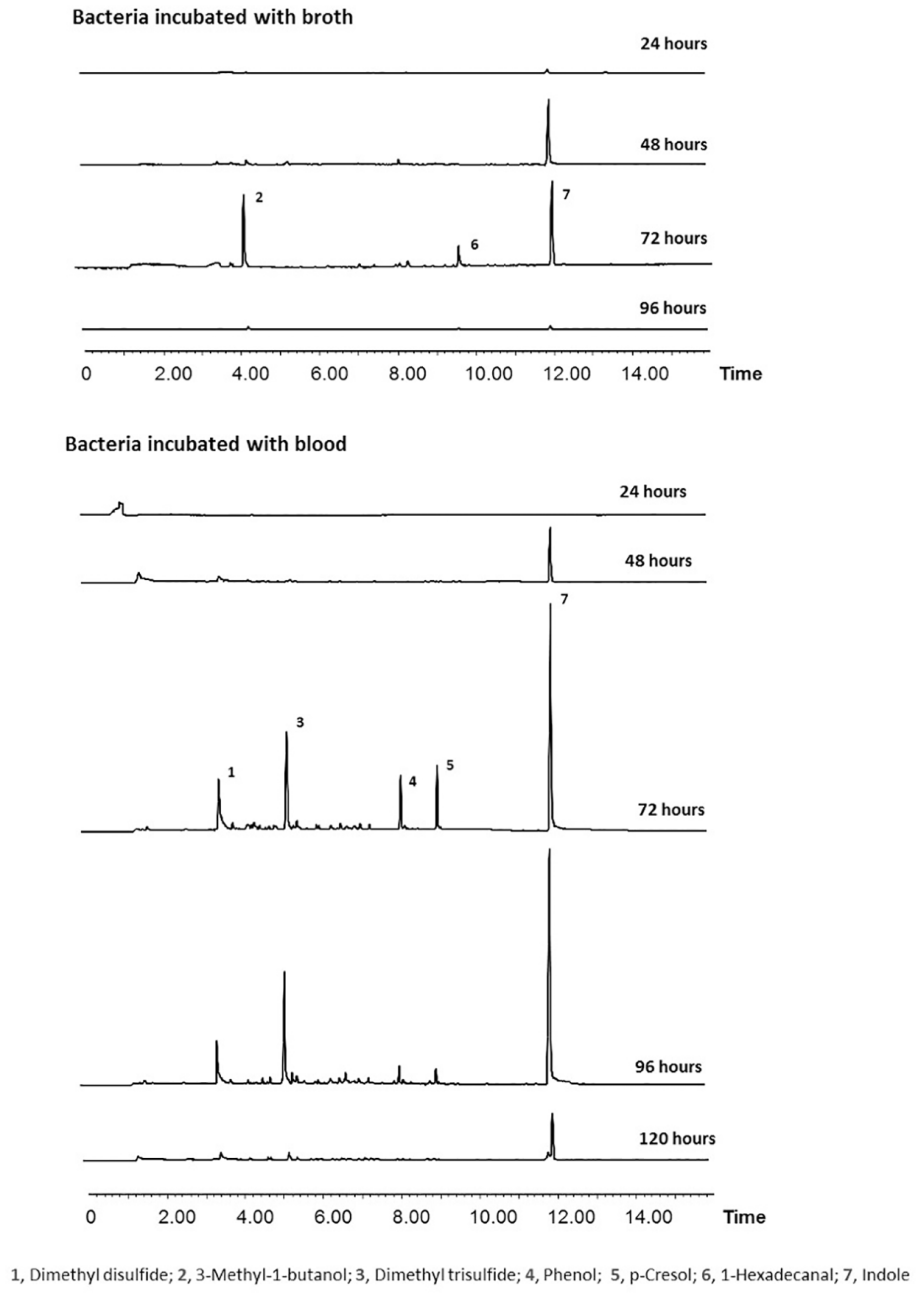
Comparisons of volatiles absorption on solid phase micro-extraction fibers of volatiles emitted from bacteria incubated with two culturing media, broth and bovine blood, with different incubation time.

As indicated by Kai et al. [33], bacterial metabolism varies in the stationary phases of different media cultures resulting in different emission. In the present study we found that the 5 bacteria mixture incubated for 72 hours released maximum levels of volatiles either in blood or broth, but the compounds released were different in proportions (Fig 2). At the 96 hour time point, the bacteria incubated in broth released almost no volatile compounds (Fig 2 upper). In contrast, the volatiles release from both the 72 hour and 96 hour-incubation in blood were very similar in both relative ratio and amount (Fig 2 lower).

Significant differences in volatiles released between a mixture of 5 bacteria and each individual bacteria incubated in blood were observed as well as those from different incubation times (Table 2). No volatiles were detected from the 5 bacteria mixture incubated in blood at the 24-hour period. However, at least 4 compounds were identified from volatiles released from four of five individual species incubated in blood, while *Proteus vulgaris* emitted five compounds. We are not sure if the five bacteria mixture needed more time to build up a population sufficient to generate the volatiles, more studies are underway to investigate the cause. Only indole was detected from volatiles absorbed in SPME fiber from the mixture of 5 bacteria species incubated for less than 48 hours. The production of indole is widespread among soil bacteria and is generated through the degradation of tryptophan by tryptophanase [24].

**Table 2:** Relative ratios of solid phase microextraction absorption (SPME) of volatile compounds released from the combined and individual five bacteria in blood at different incubation times (1–5 days).

Volatile compounds	5 species	*Pt*. *v*	*Pv*. *r*	*Pt*. *m*	*K*. *o*	*Pv*. *s*
	(*24 hours*)					
**Dimethyl disulfide**	0	0	4.19 ± 2.19	0	0	0
**Dimethyl trisulfide**	0	9.21 ± 2.20	20.84 ± 6.11	3.27 ± 2.27	3.87 ± 0.48	3.91 ± 0.57
**Phenol**	0	3.75 ± 0.11	15.98 ± 7.70	30.98 ± 2.64	5.13 ± 1.85	66.35 ± 0.39
*p***-Cresol**	0	10.49 ± 0.45	8.74 ± 2.03	9.89 ± 0.43	4.57 ± 0.92	2.86 ± 1.01
**Indole**	0	76.55 ± 2.76	50.24 ± 5.98	55.84 ± 2.79	86.43 ± 2.30	26.88 ± 1.96
	(*48 hours*)					
**Dimethyl disulfide**	0	21.58 ± 5.30	6.22 ± 3.78	18.37 ± 1.74	3.67 ± 0.35	4.46 ± 2.18
**Dimethyl trisulfide**	0	27.24 ± 1.98	24.91 ± 3.37	20.99 ± 8.12	26.61 ± 0.48	7.09 ± 1.26
**Phenol**	0	2.77 ± 1.02	29.13 ± 8.42	28.75 ± 7.85	4.47 ± 1.89	34.56 ± 0.97
*p***-Cresol**	0	4.33 ± 2.10	12.16 ± 3.35	6.45 ± 1.53	1.70 ± 0.63	12.42 ± 4.06
**Indole**	100	44.08 ± 2.04	27.57 ± 0.70	25.45 ± 5.21	63.55 ± 8.77	41.67 ± 7.31
	(*72 hours*)					
**Dimethyl disulfide**	12.52 ± 2.38	16.73 ± 4.59	8.26 ± 2.26	32.87 ± 7.49	14.02 ± 1.25	19.35 ± 6.76
**Dimethyl trisulfide**	29.94 ± 5.91	20.06 ± 1.89	11.89 ± 1.91	15.34 ± 0.98	25.70 ± 7.63	15.83 ± 5.07
**Phenol**	11.37 ± 1.76	4.33 ± 3.22	26.66 ± 2.58	6.90 ± 0.90	4.15 ± 2.25	21.92 ± 2.92
*p***-Cresol**	11.78 ± 2.26	25.77 ± 7.45	16.76 ± 7.58	17.32 ± 5.36	17.36 ± 3.83	19.81 ± 8.41
**Indole**	34.39 ± 6.44	33.10 ± 2.24	36.43 ± 1.35	27.55 ± 2.04	38.77 ± 7.29	23.07 ± 5.20
	(*96 hours*)					
**Dimethyl disulfide**	12.95 ± 3.14	12.15 ± 2.15	5.57 ± 4.50	9.28 ± 2.61	13.65 ± 1.03	10.78 ± 1.07
**Dimethyl trisulfide**	26.15 ± 8.57	19.95 ± 9.19	23.24 ± 2.61	11.14 ± 0.06	23.87 ± 3.51	18.36 ± 0.02
**Phenol**	9.84 ± 2.97	4.63 ± 2.38	26.05 ± 8.04	9.86 ± 3.86	16.93 ± 3.76	27.81 ± 0.24
*p***-Cresol**	9.13 ± 2.42	33.18 ± 7.57	26.25 ± 2.04	35.68 ± 5.28	16.37 ± 3.69	20.05 ± 1.88
**Indole**	41.84 ± 3.15	30.90 ± 7.15	18.89 ± 3.06	33.77 ± 1.60	29.17 ± 3.91	23.01 ± 1.12
	(*120 hours*) *					
**Dimethyl disulfide**	1.03 ± 0.52	0	0	0	0	0
**Dimethyl trisulfide**	0.60 ± 0.15	20.23	14.13	14.78	18.75	20.73
**Phenol**	0	4.42	12.19	5.51	3.32	24.97
*p***-Cresol**	0	25.56	51.68	28.70	27.44	31.84
**Indole**	98.37 ± 0.63	49.79	21.82	42.33	46.39	22.47
	1.03 ± 0.52	0	0	0	0	0

***Pt*. *v***. *Proteus vulgaris*; ***Pv*. *r***. *Providencia rettgeri*; ***Pt*. *m***. *Proteus mirabilis*; ***K*. *o***. *Klebsiella oxytoca*; ***Pv*. *s***. *Providencia stuartii*.

*: only one analysis was completed, the rest of samples were contaminated by the growth of mold.

Scientists have long held the view that bacterial cells behaved as self-sufficient individuals, until the discoveries of species-specific and interspecies cell-cell communication and group organization from bacterial communities [34]. Several studies have demonstrated that bacteria produce, and respond as groups to, chemical signals and that this interaction can lead to the coordination of group bacterial activities [35]. For example, Kai et al [33] reported that the growth of *Burkholderia cepacia* Yabuuchi reduces on exposure to volatiles from *Serratia odorifera* (Bizio) and *Serratia plymuthica* (Bizio). All 5 volatile compounds found in this study were found to be released from incubated individual bacteria species, and it is possible that these volatile compounds serve as a mechanism of inter-species communication between bacteria and screwworm flies. This may also be a partial explanation for the delay in volatile production; no volatiles were detected until after 24 hours, from the five bacteria mixture as indicated above.

Variations in microbial growth conditions, including incubation times, result in changes in both the types and amounts of volatiles produced [36]. From 72 to 96 hours of incubation, all 5 volatile compounds were detected from both a mixture of 5 bacteria combined and each individual bacteria, but relative ratios were different among them. Among volatiles detected, p-cresol and indole occupied 60% of total volatile compounds released from two *Proteus* bacteria while incubated alone in blood at 96-hour period. A dominant amount of indole, with over 98% of released volatiles, was detected from the 5 bacteria mixture with over 96-hour incubation time, as well as three of 5 bacteria incubated individually releasing over 40% of indole among all volatiles (*P*. *vulgaris*, *P*. *rettgeri* and *K*. *oxytoca*) (Table 2).

### Adult responses and female oviposition preferences of screwworms to the identified synthetic blend in the laboratory

**Fig 3:**
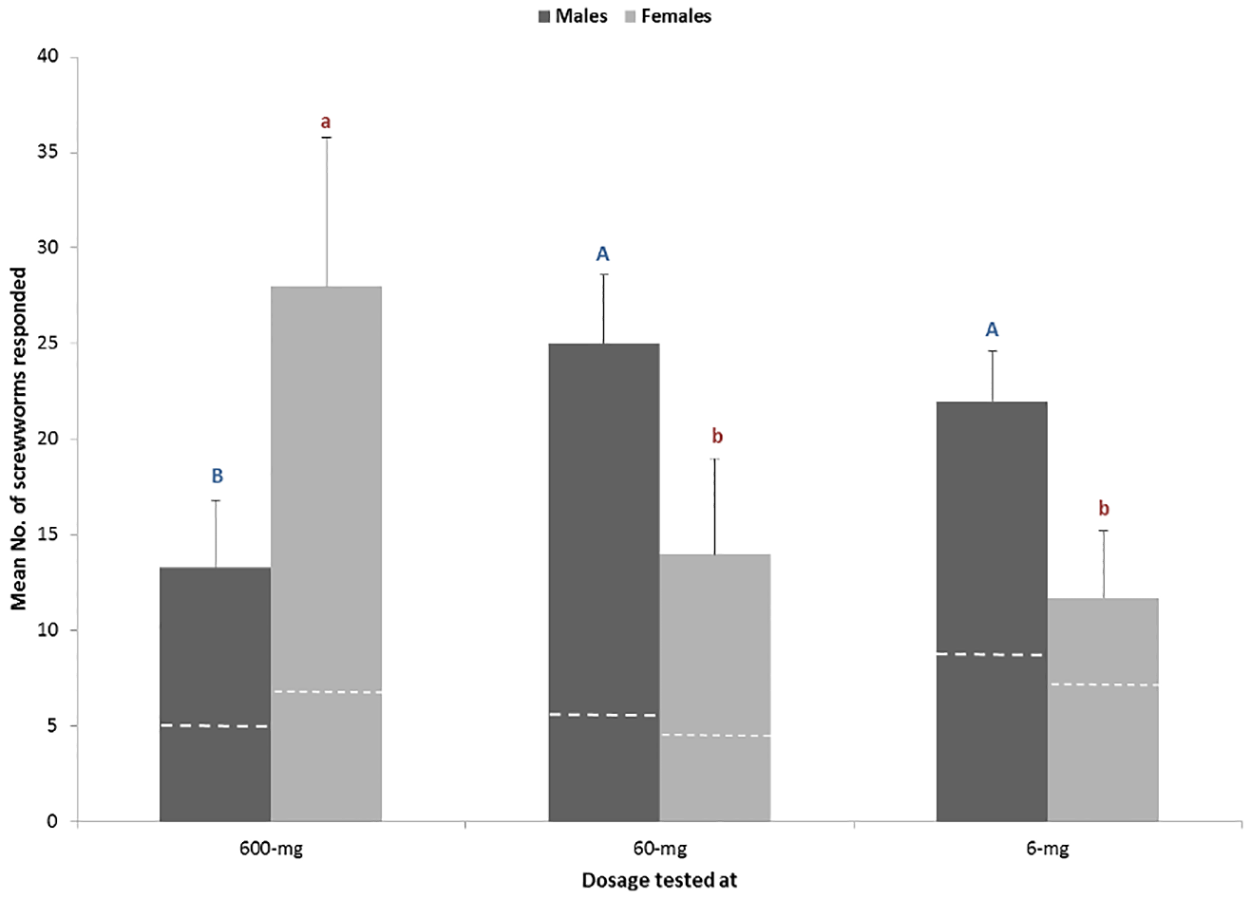
Behavioral responses of adult screwworms to the synthetic blends of a 5-component blend at three dosages of 600 mg, 60 mg and 6 mg in the laboratory assay (Mean number of screwworm flies ± Std. Error). Different letters above the bars indicate significant differences in captures of male and female flies (ANOVA, followed by LSD, P< 0.05). Dashed lines indicate mean number of flies caught from the controls.

Scanning electron microscopic observation on antennal morphology of primary screwworm flies showed several types of sensilla with wall pore structures (trichoids, basiconic and grooved coeloconica sensilla) indicating their olfactory perception of volatile substances related to their hosts and environments [37–39]. Our laboratory cage studies showed that different response profiles were found between 7-day old male and female screwworms to the tested blend at three different concentrations (Fig 3). Males showed strong responses to the two lower dosages, 6 mg and 60 mg (F = 4.30, *df* = 2, 9 P<0.05), in contrast to females which were more attracted to the highest dosage tested (600 mg) (F = 4.25, *df* = 2, 9 P<0.05). Less than 18% of primary screwworm flies responded to the control. The antagonistic effects found from males responding to the highest dosage in the current study could be due to sensory habituation, as commonly observed from other insects. However, why females responded strongly to the highest dose remains a subject for further investigation.

**Fig 4:**
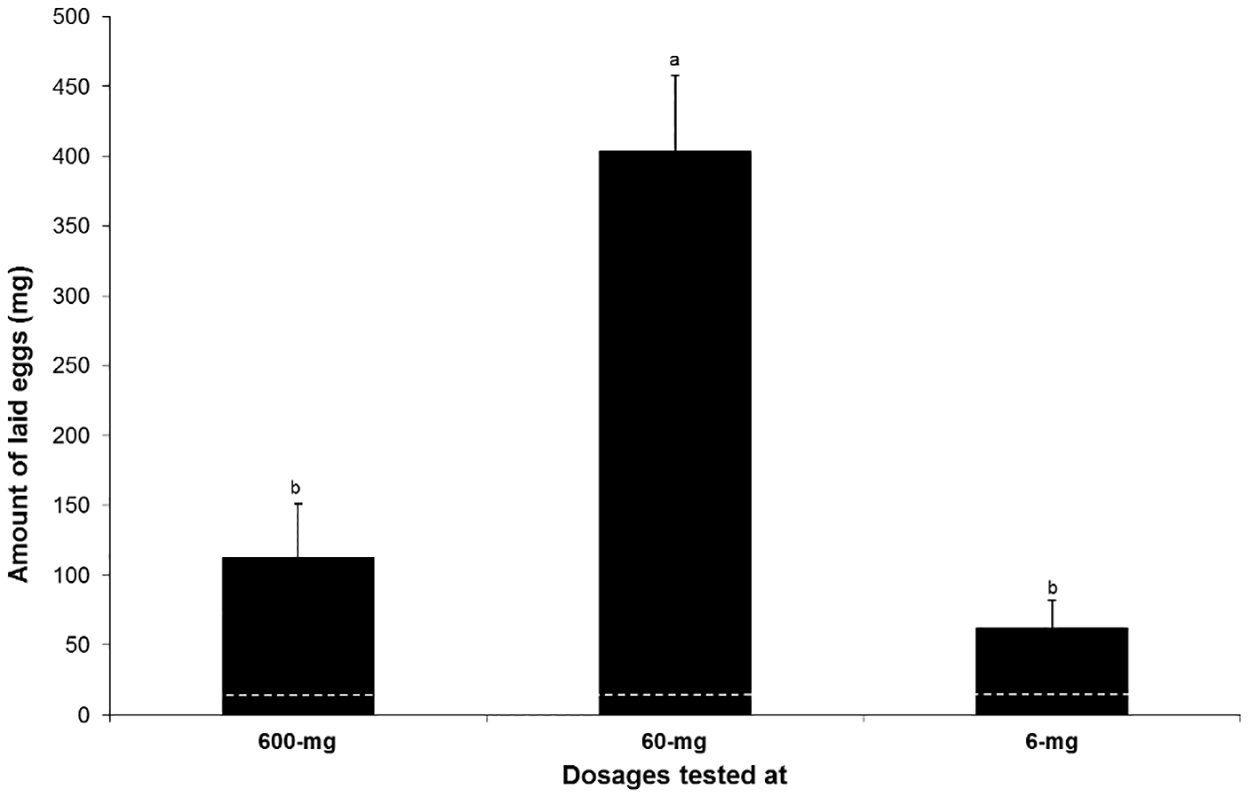
Amount of eggs (Means ± Std. Error) laid by gravid females of primary screwworm flies tested in cages baited with synthetic lures at three dosages of 600 mg, 60 mg and 6 mg. Different letters on top of the bars indicate significant differences (ANOVA, followed by LSD, P< 0.05). Dashed lines indicate mean amount of eggs laid from the controls.

Chaudhury et al. [20] reported that Bovine blood inoculated with eight bacteria isolated from primary screwworm-infested animal wounds is attractive to adult screwworms. They further demonstrated that bovine blood incubated with 5 bacteria (*Klebsiella oxytoca*, *Proteus mirabilis*, *Proteus vulgaris*, *Providencia rettgeri*, and *Providencia stuartii*) attracted more females than those of three other species [*Enterobacter cloacae* (Jordan), *Cronobacter* (*Enterobacter) sakazakii* (Farmer et al.), and *Serratia liquefaciens* (Grimes and Hennerty)], with more egg deposition observed from 72-hr incubation. We also found that significantly more eggs were laid on the oviposition media baited with the 5-component blend at a ratio equivalent to that from 72 hours’ incubation (T-test, t = 2.35–3.18, P<0.05). This was observed at all three doses. The highest amount of eggs (403 mg) were received from media with a 60 mg dosage, compared to 112 mg and 62 mg egg harvested from media baited with 600mg and 6 mg, respectively (Fig 4). Less than 10 mg of eggs were received on the control media. The highest egg deposition found from this study is similar to what has been reported from Chaudhury et al. [20], although they incubated eight bacteria.

**Fig 5:**
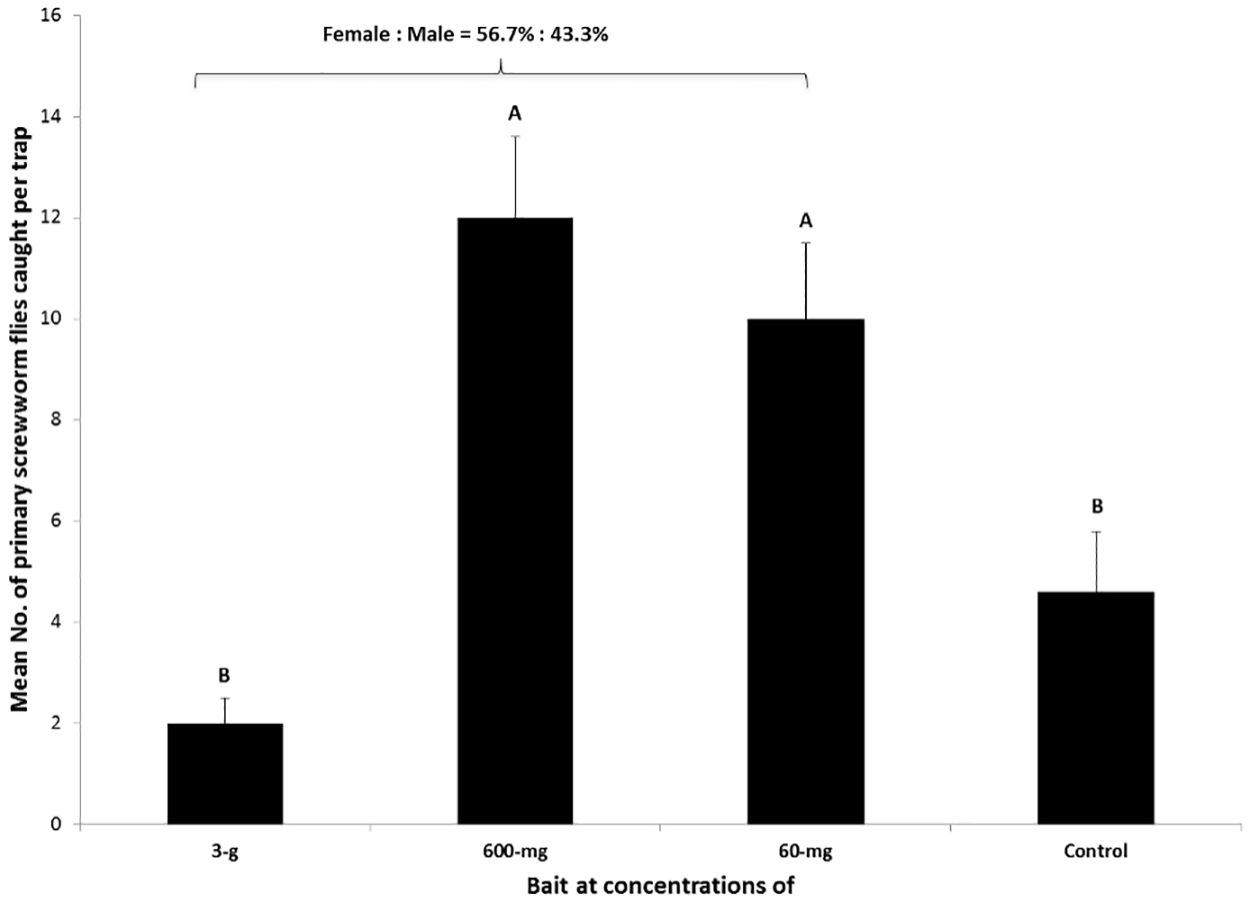
Mean number of primary screwworm flies (Means ± Std. Error) caught in traps baited with synthetic lures at dosage of 3 g, 600 mg and 60 mg. Different letters on top of bars indicate significant differences (ANOVA, followed by LSD, P< 0.05).

Field trials conducted in Brazil, where primary screwworm flies were present at a local hog farm, showed that traps baited with 600 mg and 60 mg of the identified 5-component blend caught significantly more flies, compared to the 5X concentrated bait and the control. (Fig 5, F = 9.386, df = 3, 25; P <0.001). The mean sex ratio among captured screwworm flies from all treatments was 56.7 ± 4.1% of females: 43.3 ± 4.0% of males, which indicated that the blend was equally attractive to primary screwworm flies of both sexes in the field (Student T-test, t = 1.56–2.57, P>0.05). The results from the field trapping tests were similar to what was observed in other field observations from other South American countries (unpublished). In the laboratory, cage study bioassays also showed that the highest doses tested displayed antagonistic effects on male flies. Females in the laboratory seemed more attractive to the highest dosage (600 mg). The same dosage tested in the field trial also showed the highest number of flies caught in the trap in both sexes, with a similar level of attraction to flies from traps baited with a lower dosage at 60 mg. In contrast, traps baited with this lower dose caught significant less number of female flies, but more males in the laboratory assay. Such a discrepancy remains a subject for further investigation. The highest dose at 3 g tested in the field trial acted antagonistically in attraction to adult flies.

The 5-component blend identified from the blood inoculated with a mixture of 5 bacteria isolated from the animal wounds infested by the primary screwworm is somewhat similar to those found from the spent diet from the screwworm larval rearing media [14]. However, the relative ratios of the five components from SPME absorption are different [14]. The relative ratio of dimethyl disulfide, dimethyl trisulfide, phenol, p-cresol and indole from the spent diet is 53.6%: 35.2%: 9.1%: 0.2%: 1.9%, compared to the present study from emissions of the bovine blood inoculated with 5 bacteria at 18.1%: 22.6%: 16.1%: 10.2%: 33%, respectively. Cork [25] isolated about 26 electrophysiologically-active compounds from sheep wounds infested with larvae of primary screwworms. Among them, phenol and indole were the only two compounds found from our SPME collection from the blood incubated with the 5 bacteria mixture. The 5-component synthetic blend has been demonstrated to elicit strong egg deposition from both secondary screwworms, *C*. *macellaria* and primary screwworms, but only under laboratory conditions [14] and present study. In the field, a ten-compound swormlure-4 blend was attractive to males and females of *C*. *hominivorax*, although with only three of the ten compounds similar to what was identified from the present study [40, 41].

Chaudhury et al. [19] demonstrated that the blood inoculated for 72 hours with 8 bacteria species isolated from screwworm-infested animal wounds elicited significant responses from female primary screwworms, as well as their egg deposition. Our laboratory study also showed that the synthetic blend containing 5 compounds at the ratio from SPME collection of the blood inoculated with the bacteria mixture at 72 and 96 hours strongly stimulated the primary screwworm egg deposition. The releases of these 5 volatile compounds at 24-, 48- and 120 hours were significantly different from 72- and 96-hour incubation periods. However, whether differences in release ratios between 72- and 96-hour incubation times have the same attractivity in behavioral responses and egg deposition to primary screwworms in the field needs to be further studied, as well as the efficacy of different synthetic blends (including sub-blend compositions) performance in the field.

## Conclusions

This is the first study that reports different volatile release patterns found in individual and a mixture of bacteria species incubated with blood. These differences are possibly due to different bacteria-derived byproducts that may serve as precursors of released volatile compounds, with further complications that growth competitions among multi-species in this bacterial community may prevent or delay the release of some volatiles. However, the outcomes from such studies could be used to improve the attractiveness and specificity of synthetic baits used for monitoring and sampling primary screwworm field populations, contributing to the development of a more effective surveillance tool that detects outbreaks at very early stages and control strategies to reduce the incidence of myiasis in livestock and wildlife in their extant range.
